# Preparation and Characterization of PCL/PEO-PVP ECM-Mimicking Coaxial Electrospun Membranes Loaded with Ciprofloxacin and Curcumin for Sequential Dual-Drug Release

**DOI:** 10.3390/biomimetics11080599

**Published:** 2026-08-21

**Authors:** Haiguang Zhang, Feng Jiang, Qianmin Gao, Qingxi Hu, Jiaxuan Feng

**Affiliations:** 1 Rapid Manufacturing Engineering Center, School of Mechatronic Engineering and Automation, Shanghai University, Shanghai 200444, Chinahuqingxi@shu.edu.cn (Q.H.); 2 National Demonstration Center for Experimental Engineering Training Education, Shanghai University, Shanghai 200444, China; 3 Shanghai Key Laboratory of Intelligent Manufacturing and Robotics, Shanghai University, Shanghai 200444, China; 4 Institute of Translational Medicine, Shanghai University, Shanghai 200444, China; 5 Vascular Surgery Department, Ruijin Hospital, Shanghai Jiaotong University School of Medicine, Shanghai 200025, China

**Keywords:** coaxial electrospinning, ciprofloxacin, curcumin, sequential drug release, vascular stent coating

## Abstract

Vascular stent implantation is a major treatment for vascular diseases, yet postoperative infection and persistent inflammation increase the risk of in-stent restenosis. Herein, core–shell structured PCL/PEO-PVP fiber membranes co-loaded with ciprofloxacin hydrochloride (CIP) and curcumin (CUR) were fabricated via coaxial electrospinning. Orthogonal experiments were conducted to optimize critical spinning parameters through multi-index comprehensive evaluation. Characterizations confirm intact core–shell architecture and stable polymeric backbone structure. In vitro release tests reveal sequential drug-delivery behavior: a rapid initial release of hydrophilic CIP and a delayed sustained release of hydrophobic CUR were observed, contributing to early-stage antibacterial and long-term anti-inflammatory effects, respectively. The “antibacterial zone” method verifies favorable antibacterial activity against *Staphylococcus aureus* (*S. aureus*) and *Escherichia coli* (*E. coli*). ELISA results demonstrate the enhanced anti-inflammatory capacity of the dual-drug-loaded coaxial fiber membrane. Cellular assays confirm satisfactory cytocompatibility, and endothelial cells achieve normal proliferation and exhibit typical polygonal morphology on the membrane surface. This dual-drug-loaded coaxial-fiber membrane realizes coordinated sequential antibacterial and anti-inflammatory properties, which provides a feasible strategy for developing functional coatings toward vascular stents.

## 1. Introduction

Blood vessels are essential components of the human circulatory system, possessing a unique multilayered tubular structure and performing core physiological functions including blood transportation and substance metabolism [1]. Various vascular lesions disrupt normal blood circulation and trigger numerous complications, posing a serious threat to human health [1,2]. With the rapid development of endovascular interventional medicine, vascular stent implantation has become a major clinical strategy for treating hemangiomas and vascular stenosis [3]. Nevertheless, sustained chronic inflammation at the implantation site remains an intractable clinical challenge following stent deployment. This inflammation easily induces abnormal intimal hyperplasia of blood vessels, ultimately resulting in in-stent restenosis and greatly compromising the long-term therapeutic efficacy of stents [4,5,6]. In addition, bacterial infection is another non-negligible concern. Despite its relatively low overall incidence, infection may provoke severe local and systemic adverse reactions. In severe cases, secondary open debridement surgery is required, which causes substantial secondary trauma and imposes an increased medical burden on patients [7,8,9].

In recent years, drug-loaded coatings for vascular stents have emerged as a promising strategy to alleviate these complications. By incorporating multiple drugs or bioactive agents, such coatings can realize early antibacterial effects and long-term anti-inflammatory functions, overcoming the performance limitations of conventional single-drug-eluting stents [10,11,12]. Ciprofloxacin (CIP) is a broad-spectrum quinolone antibiotic with potent inhibitory activity against both Gram-positive and Gram-negative bacteria, and it is widely utilized in clinical practice to prevent and treat infections following interventional procedures [13,14]. Ciprofloxacin hydrochloride (CIP·HCl, hereinafter abbreviated as CIP) exhibits favorable water solubility, rapid onset of action, stable antibacterial efficacy, and low cytotoxicity both in vitro and in vivo. It satisfies the requirements for drug loading in biomedical implant materials and can be rapidly released upon exposure to body fluids to eliminate local pathogenic bacteria in a timely manner [15]. Curcumin (CUR) is a natural polyphenol extracted from Zingiberaceae plants, featuring abundant raw material sources, favorable biosafety and negligible systemic cytotoxicity. Numerous studies have verified that curcumin can downregulate the expression of inflammatory cytokines and simultaneously exert moderate antibacterial activity [16,17]. Benefiting from its prominent biological functions, including anti-inflammation, antioxidation and suppression of excessive vascular smooth muscle cell proliferation, CUR has been extensively explored for developing vascular implant biomaterials [18,19]. Nevertheless, free curcumin suffers from extremely poor water solubility, facile oxidative degradation under physiological conditions, and rapid systemic clearance. These drawbacks lead to short retention duration at the implantation site, rendering it incapable of maintaining sustained anti-inflammatory and anti-proliferative effects for weeks after implantation. Therefore, constructing a suitable polymeric drug delivery carrier for efficient co-encapsulation of the two drugs constitutes the core scientific issue addressed in this study.

A variety of carrier systems have been developed to enhance drug stability and tailor drug release profiles, including drug-loaded microspheres, degradable hydrogels and polymeric coating films [20,21,22]. Electrospun fibers have attracted extensive research interest as implantable drug-loaded stent coatings, thanks to their biomimetic porous network structure mimicking native extracellular matrix, ultrahigh specific surface area and tunable pore structure [23]. The electrospinning technique enables relatively facile fabrication of continuous fibers. Their interconnected porous architecture facilitates fluid permeation, nutrient transport and cell adhesion, making electrospun fibers an ideal platform for encapsulating small-molecule drugs and precisely regulating drug release behavior. Among diverse electrospinning techniques, coaxial electrospinning adopts a dual-channel independent feeding system to fabricate core–shell structured fibers. Drugs can be encapsulated within the core layer, which effectively avoids adverse effects induced by initial burst release arising from direct contact between drugs and the physiological environment. Although many drug-loaded vascular coatings have been prepared via coaxial electrospinning, most of them only load a single drug and fail to achieve sequential drug delivery; multi-layer stacked structures are generally required for multi-drug loading [24,25]. Accordingly, this study employs coaxial electrospinning to co-load CIP and CUR inside fiber cores. Taking advantage of their distinct physicochemical properties, sequential drug release can be achieved, allowing the coating to deliver early intensive antibacterial activity and persistent long-term anti-inflammatory performance.

Polycaprolactone (PCL) is an FDA-approved synthetic polymer widely adopted in drug delivery systems and tissue engineering. It possesses desirable mechanical properties and biocompatibility to meet the fundamental mechanical and structural demands of vascular stent coatings [26]. Polyethylene oxide (PEO) and polyvinylpyrrolidone (PVP) are water-soluble biomedical polymers characterized by high hydrophilicity and low cytotoxicity [27,28]. PEO acts as the primary drug carrier with excellent spinnability and appropriate mechanical properties, whereas PVP serves as an auxiliary drug carrier to disperse and stabilize drugs, adjust the rheological properties of spinning solutions, and further improve electrospinnability.

In this work, hydrophobic PCL was selected as the fiber shell layer, while a hydrophilic PEO/PVP blend system served as the core matrix for co-encapsulating CIP and CUR. Key coaxial electrospinning parameters, including spinning solution concentration, core-to-shell flow rate ratio, applied voltage and collecting distance, were systematically optimized to prepare uniform, bead-free PCL/PEO-PVP@CIP@CUR core–shell dual-drug fiber membranes. Comprehensive physicochemical characterizations were performed to evaluate micromorphology, chemical structure, wettability and mechanical properties. The in vitro sequential drug-release profiles and antibacterial and anti-inflammatory effects were investigated. Finally, human umbilical vein endothelial cells (HUVECs) were adopted for cytocompatibility evaluation. This study aims to verify the potential of the PCL/PEO-PVP@CIP@CUR electrospun fiber membrane as a functional coating for vascular stents.

## 2. Materials and Methods

### 2.1. Materials

Polycaprolactone (PCL, molecular weight: 80–100 kDa) and curcumin (CUR) were purchased from Aladdin (Shanghai, China). Dichloromethane (DCM) and *N,N*-dimethylformamide (DMF) were supplied by Sinopharm Chemical Reagent Co., Ltd. (Shanghai, China). Ultrapure water and lipopolysaccharide (LPS, O111:B4) were obtained from Merck Chemical Technology (Shanghai, China) Co., Ltd. Absolute ethanol, ciprofloxacin hydrochloride, and polyethylene oxide (PEO, molecular weight: 10 kDa) were purchased from Macklin (Shanghai, China). PVP K60 (molecular weight: 22 kDa) was obtained from Foshan Yike Chemical Reagent Co., Ltd. (Foshan, China). The RAW 264.7 cell line (RRID: CVCL_0493, Procell catalog No. CL-0190) was obtained from Procell Life Science & Technology Co., Ltd. (Wuhan, China). The HUVEC cell line (RRID: CVCL_2959, catalog No. SCSP-5330) was obtained from the Cell Bank of the Chinese Academy of Sciences (Shanghai, China). All experiments were performed in vitro using established cell lines.

### 2.2. Preparation of Electrospinning Solutions

First, the shell spinning solution was prepared. For core–shell coaxial fibers, the shell material serves as the critical component to sustain overall fiber morphology and maintain the mechanical stability of the membrane, directly governing the structural strength and deformation resistance of the vascular stent coating. Accordingly, PCL with favorable biocompatibility, stable mechanical performance, and a degradation cycle matching vascular tissue regeneration was selected as the shell substrate. A DCM/DMF mixed solvent with a volume ratio of 7:3 was prepared at room temperature. Precisely weighed PCL pellets were dissolved in the mixed solvent, followed by continuous sealed magnetic stirring at room temperature for 4 h to yield a homogeneous and transparent PCL shell spinning solution at a concentration of 15% (*w*/*v*).

Unlike the shell layer, which is primarily designed for mechanical support, the core layer of coaxial fibers undertakes drug loading and controlled drug release. It requires balanced hydrophilicity, cytocompatibility and drug dispersion stability, which cannot be realized using a single polymer. In this study, a blended PEO/PVP polymer system was adopted to construct the core matrix. The synergistic effect of the two polymers optimizes the rheological properties of the spinning solution and provides a suitable matrix for dual-drug loading and sequential drug release.

**Figure 1:**
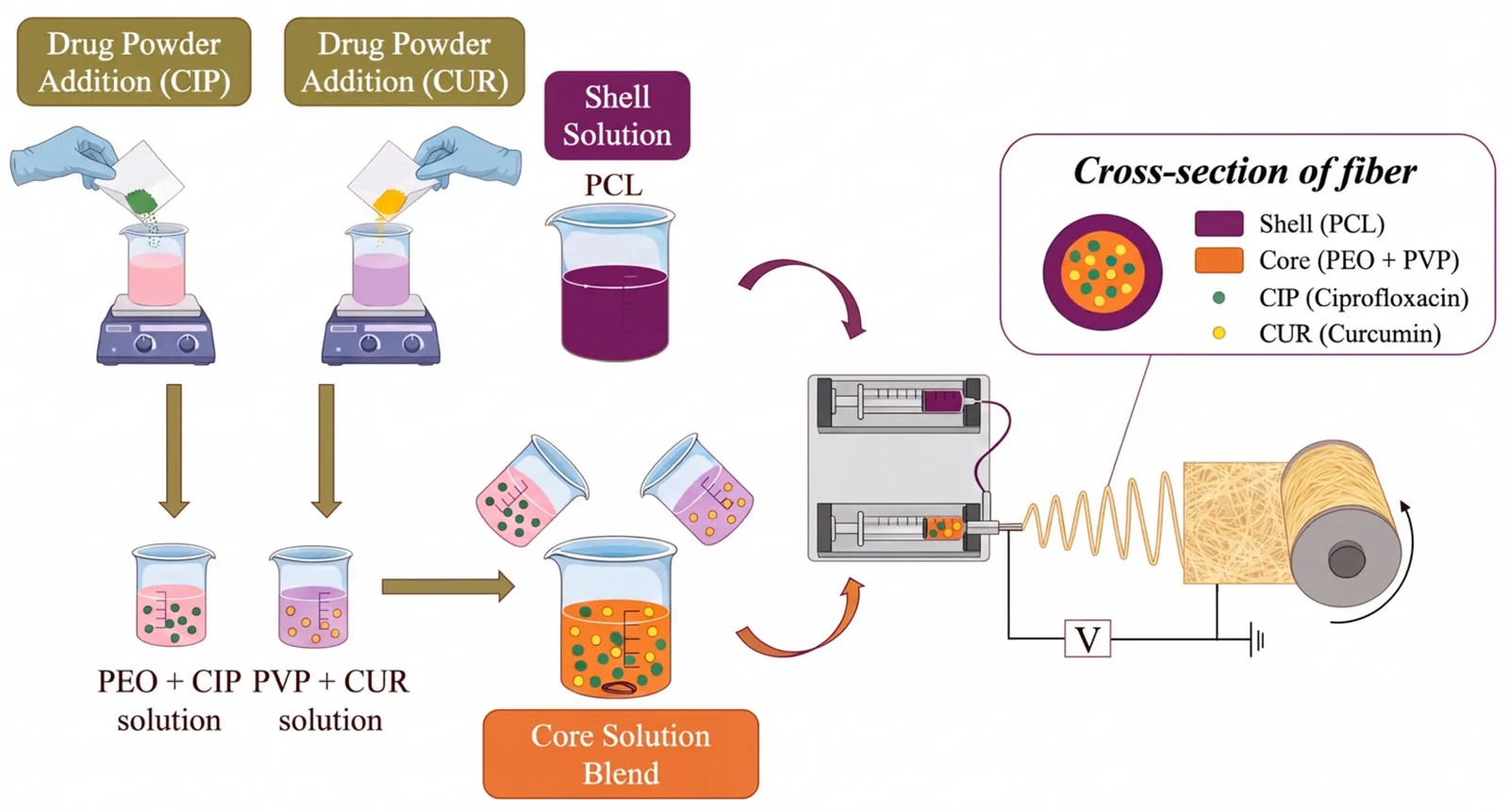
Fabrication of dual-drug loaded coaxial electrospun fibrous membranes.

CIP features favourable hydrophilicity and can dissolve rapidly in water, while CUR is hydrophobic, insoluble in water, slightly soluble in ethanol and highly soluble in DMF. In addition, an ethanol-aqueous mixed solvent is required for the satisfactory dissolution of PEO and PVP. Accordingly, a mixed-solvent system was designed. The core solvent consisted of ethanol, ultrapure water and DMF at a volume ratio of 6:3:1. PEO powder and CIP powder were sequentially added into an ethanol/water mixture (volume ratio 3:2) and stirred until fully dissolved. Separately, PVP pellets and CUR powder were added to an ethanol/water/DMF mixture (volume ratio 3:1:1) and stirred to complete dissolution. The two solutions were prepared with identical drug concentrations, and the mass ratio of PEO to PVP was fixed at 3:1. The two resultant solutions were blended at a volume ratio of 1:1 and further homogenized via stirring. All stirring operations for the core solution were conducted under sealed and light-shielded conditions. Finally, a series of PEO/PVP dual-drug-loaded core spinning solutions were fabricated with total polymer concentrations of 2% (*w*/*v*), 4% (*w*/*v*), 6% (*w*/*v*) and 8% (*w*/*v*); both drugs were kept at a constant concentration of 2 mg/mL. The detailed preparation process is displayed in Figure 1.

### 2.3. Orthogonal Experiment

#### 2.3.1. Determination of Key Coaxial Electrospinning Parameters and Factor Levels

The primary parameters influencing coaxial electrospinning include solution concentration, core-to-shell flow rate ratio, electrospinning voltage, collecting distance, ambient humidity, etc. In this study, ambient humidity was stabilized at approximately 45% using a dehumidification system to create a favorable electrospinning environment. A 15% (*w*/*v*) PCL shell solution has been extensively verified in previous work for producing continuous and uniform fibers [29,30,31]; therefore, the shell concentration was fixed at 15% (*w*/*v*). Accordingly, four factors were chosen for the orthogonal experiment: total core solution concentration (A), core-to-shell flow rate ratio (B), electrospinning voltage (C), and collecting distance (D).

Single-variable experiments were first carried out to explore the effect of each parameter on fiber morphology, while the remaining parameters were maintained constant. The tested total core solution concentrations were 2% (*w*/*v*), 4% (*w*/*v*), 6% (*w*/*v*) and 8% (*w*/*v*); core-to-shell flow rate ratios were 1:3, 1:4, 1:5, 1:6 and 1:7 (the total feeding flow-rate was kept constant); electrospinning voltages were 10 kV, 12 kV, 14 kV and 16 kV; collecting distances were 12 cm, 15 cm, 18 cm and 21 cm. According to the macroscopic and micromorphological characteristics of the obtained fiber membranes, three suitable levels were determined for each factor to construct a four-factor, three-level orthogonal experimental design.

#### 2.3.2. Orthogonal Experimental Design and Evaluation Criteria

In accordance with the practical requirements for vascular stent coatings, four evaluation indices were employed for multi-factor process optimization.

- The 4 h cumulative release rate of curcumin (*a*) was adopted to evaluate the early burst release behavior, aiming to realize long-term sustained anti-inflammatory therapy;
- The tensile strength of the fiber membrane (*b*) was measured to characterize the mechanical structural stability of the material;
- The coefficient of variation in fiber diameter (*c*) was used to assess fiber uniformity, so as to guarantee consistent batch-to-batch performance;
- The total drug loading content (*d*) was determined to quantitatively evaluate the overall drug loading capacity and avoid inadequate drug loading induced by extreme core-to-shell flow rate ratios.

All four indices were dimensionless-normalized within the range of experimental data and denoted as *y_a_*, *y_b_*, *y_c_*, and *y_d_*. A comprehensive score y was calculated to realize synergistic optimization covering sustained drug release, mechanical properties, fiber uniformity and drug loading capacity. The calculation formula is expressed as follows:

(1) $$y_{a}=\frac{a_{max}-a}{a_{max}-a_{min}}$$

(2) $$y_{b}=\frac{b-b_{min}}{b_{max}-b_{min}}$$

(3) $$y_{c}=\frac{c_{max}-c}{c_{max}-c_{min}}$$

(4) $$y_{d}=\frac{d-d_{min}}{d_{max}-d_{min}}$$

(5) $$y=10\times \left(0.35y_{a}+0.3y_{b}+0.2y_{c}+0.15y_{d}\right)$$

The sum of the scores for the same level of each of the four factors A, B, C and D is denoted as *K*_1_, *K*_2_, *K*_3_, and the corresponding average values are denoted as *k*_1_, *k*_2_, *k*_3_. The calculation formulas are as follows:

(6) $$K_{i}=\sum_{t=1}^{n}y_{x_{i},t}$$

where x = 1, 2, 3, 4 corresponds to the four factors in sequence, and i = 1, 2, 3 corresponds to the three levels.

(7) $$k_{i}=\frac{K_{i}}{n}$$

where n refers to the number of experimental replicates for the same level of a given factor, and n = 3 in this experimental scheme.

*k*_max_ and *k*_min_ represent the maximum and minimum scores of the same factor across different levels, respectively. The range value *R* is defined as their difference, which is used to evaluate the influence degree of each factor on the experimental results. The calculation formula is as follows:

(8) $$R=k_{max}-k_{min}$$

The optimal combination of process parameters was determined based on the comprehensive scores, and the primary and secondary influencing orders of the parameters were identified by comparing the range value *R* of each factor, wherein a larger *R*-value represents a stronger influence of the corresponding factor. The dual-drug coaxial fiber membrane fabricated using the optimal process parameters was designated PCL/PEO-PVP@CIP@CUR for subsequent evaluations of physicochemical properties, antibacterial and anti-inflammatory performance, and biosafety.

### 2.4. Microscopic Morphology Characterization

Electrospun fiber membranes were cut into suitable dimensions and securely mounted onto scanning electron microscopy (SEM) stubs with conductive adhesive. Subsequently, samples underwent gold sputtering for conductive surface coating, and the thickness of the gold layer was controlled within 10–15 nm. The treated specimens were examined with a Hitachi SU-1500 scanning electron microscope (Hitachi, Tokyo, Japan) at an appropriate accelerating voltage to characterize surface micromorphology, capture micrographs, and analyze fiber diameter distribution.

Transmission electron microscopy (TEM) was utilized to visualize the core–shell architecture of fibers. While wearing non-conductive dust-free gloves, a 200-mesh carbon-coated copper grid was held and swept gently and uniformly at a distance of 15 cm away from the coaxial electrospinning nozzle, enabling fibers to deposit naturally onto the carbon film. The fiber-carrying copper grid was observed using a JEM-2100 transmission electron microscope (JEOL, Tokyo, Japan), and TEM micrographs were obtained to directly verify the layered core–shell structure.

### 2.5. Mechanical Properties

**Figure 2:**
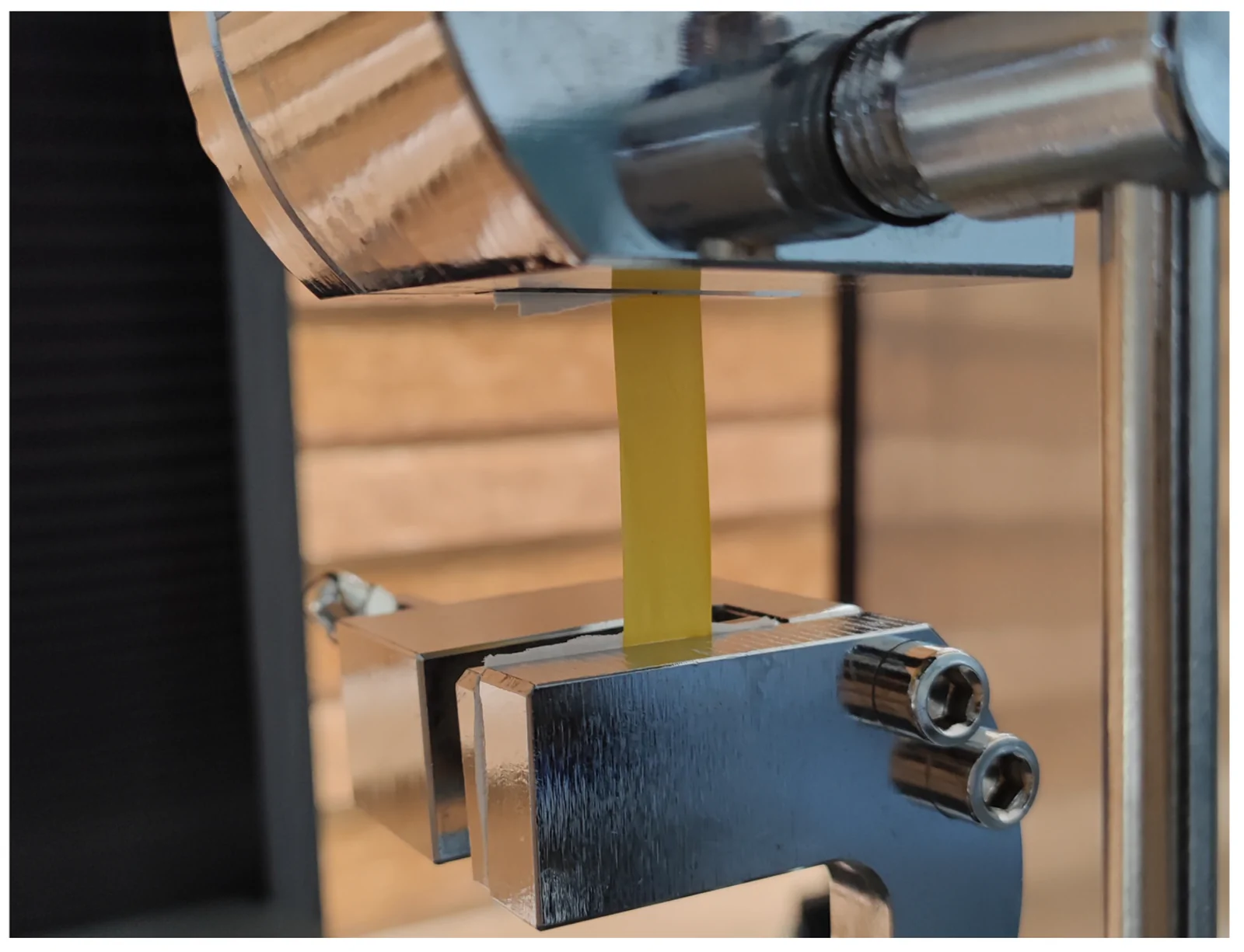
Experimental setup for uniaxial tensile measurement of electrospun fibrous membranes.

As shown in Figure 2, Tensile mechanical measurements were performed on a WDW-1 universal material testing machine (Songton, Shanghai, China). The fiber membranes were cut into rectangular specimens with dimensions of 60 mm × 10 mm. The thickness of each specimen was measured at five locations using a digital micrometer, and the average value was recorded. Uniaxial longitudinal tensile tests were conducted at room temperature with a crosshead speed of 10 mm/min. Three parallel replicates were tested for each group (n = 3), and the data sampling frequency was set to 10 Hz.

### 2.6. Hydrophilicity Measurement

The water contact angle (WCA) was measured using a JC2000D1 contact angle goniometer (Powereach, Shanghai, China) to assess the surface wettability of the fiber membranes. Specimens (30 mm × 10 mm) were fixed flat onto glass slides and placed on the instrument stage. A 4 μL ultrapure water droplet was deposited onto the membrane surface, and high-resolution droplet images were captured within 30 s for contact angle calculation. Five parallel measurements were conducted for each sample (n = 5), and the average value was reported.

### 2.7. FTIR Characterization

Attenuated total reflection Fourier-transform infrared spectroscopy (ATR-FTIR) was carried out on a Nicolet AVATAR370 spectrometer (Thermo Fisher, Waltham, MA, USA) to verify the chemical structural stability of polymer raw materials before and after electrospinning and characterize the backbone chemical structure of fiber membranes. Before measurement, all samples, including raw PEO, PVP powders, pure PCL fiber membranes, and PCL/PEO-PVP@CIP@CUR dual-drug coaxial fiber membranes, were vacuum-dried to remove residual solvent, and each dried specimen was closely attached to the ATR crystal for direct detection. The spectral resolution was set to 1 cm^−1^, and the scanning wavenumber range was 4000–600 cm^−1^. Infrared spectra were acquired to analyze variations in characteristic functional groups before and after electrospinning.

### 2.8. Degradation Test

The degradation behavior of fiber membranes (including PCL/PEO-PVP@CIP@CUR) was assessed via gravimetric measurement. Three pre-cut membrane specimens (n = 3) were fully dried and weighed to record the initial mass W_0_. Subsequently, the specimens were completely immersed in phosphate-buffered saline (PBS, pH = 7.4) and incubated at 37 °C. Specimens were retrieved at predetermined time points (1 h, 2 h, 4 h, 8 h, 12 h, 1 d, 2 d, 3 d, 5 d, 1 week, 2 weeks), dried to constant weight at 37 °C, and weighed to obtain the residual mass W_t_. After weighing, the specimens were placed back into PBS for continuous incubation, and this procedure was repeated for subsequent time intervals. The formula used to calculate the mass loss rate at each time point is given below:

(9) $$\mathrm{mass}\;\mathrm{loss}\;\mathrm{rate}=\frac{W_{0}-W_{t}}{W_{0}}\times 100\%$$

### 2.9. Drug Loading and in Vitro Release Behavior

#### 2.9.1. Drug Absorbance Measurement

To quantify drug concentrations in the solution, the absorbance (Abs) of sample extracts was measured with a 752 UV spectrophotometer (Jinghua, Shanghai, China). Absorbance readings were acquired at 277 nm and 425 nm, corresponding to the maximum absorption wavelengths of CIP and CUR, respectively [32,33]. The standard absorbance–concentration calibration curves of the two drugs at these wavelengths were adopted to calculate drug concentrations in the extracts. CUR displays weak absorbance at 277 nm, which introduces slight interference to the CIP absorbance measurement; such interference was subtracted to obtain the net absorbance of CIP. In comparison, CIP exhibits negligible absorbance at 425 nm, and thus the measured absorbance directly represents the net absorbance of CUR.

#### 2.9.2. Actual Drug Loading Content

The drug loading content was determined to characterize the drug loading performance of PCL/PEO-PVP@CIP@CUR fiber membranes. Membrane specimens (50 mm × 50 mm, approximately 50 mg) were fully immersed in 25 mL of 50% ethanol solution and shaken on an orbital shaker at 180 r/min for complete drug extraction. A 5 mL aliquot of the supernatant was withdrawn for absorbance measurement every 2 h until the absorbance value remained constant (typically after 2–3 measurements). The absorbance data were adopted to calculate the actual loaded amounts of CIP and CUR. Combined with the total mass of the fiber membrane, the total drug loading content *d* described in Section 2.3.2 was calculated [34]:

(10) $$d=\frac{m_{\mathrm{CIP}}+m_{\mathrm{CUR}}}{m_{\mathrm{mem}}}\times 100\%$$

#### 2.9.3. Cumulative in Vitro Drug Release Profile

To explore the in vitro drug release profiles of PCL/PEO-PVP@CIP@CUR fiber membranes, specimens (50 mm × 50 mm) were fully immersed in 25 mL of PBS (pH = 7.4) supplemented with 0.1% Tween 80. Samples were incubated in an orbital shaker at 37 °C and 75 r/min to mimic the in vivo release microenvironment. At predetermined time points (0.5 h, 1 h, 2 h, 4 h, 8 h, 12 h, 24 h, 36 h, 48 h, 72 h, 96 h, 120 h, 144 h, 168 h), 5 mL of supernatant was withdrawn for absorbance measurement to calculate drug concentrations and plot release curves. An equal volume of fresh medium was replenished following each sampling to keep the total volume constant.

### 2.10. Antibacterial Performance Evaluation

The “antibacterial zone” method was adopted to evaluate the antibacterial efficacy of dual-drug coaxial fiber membranes against representative Gram-positive *Staphylococcus aureus* (*S. aureus*) and Gram-negative *Escherichia coli* (*E. coli*). This assay relies on the diffusion of soluble antibacterial agents released from fiber mats into surrounding agar medium to form bacteria-free transparent inhibition regions. The two bacterial strains were cultured in Luria–Bertani (LB) liquid medium to prepare bacterial suspensions at a concentration of approximately 10^8^ CFU/mL. Square membrane specimens (10 mm × 10 mm) were placed onto LB agar plates uniformly spread with 100 μL of bacterial suspension. Each group was performed with at least three independent experimental replicates. Following incubation at 37 °C for 24 h, the plates were photographed under identical lighting conditions. The relative antibacterial performance of different groups was qualitatively compared by observing and comparing the size and clarity of inhibition zones; generally, a larger clear inhibition zone indicates relatively stronger antibacterial activity.

### 2.11. Anti-Inflammatory Performance Evaluation

Enzyme-linked immunosorbent assay (ELISA) was conducted to detect key inflammatory cytokines (TNF-α, IL-6) and assess the anti-inflammatory effects of the samples. RAW264.7 murine macrophages were employed, and lipopolysaccharide (LPS) was utilized to trigger inflammatory responses. Cells were seeded in 24-well culture plates and incubated overnight to achieve complete adherence. The original culture medium was subsequently replaced, and Transwell inserts were placed into each well; square fiber membrane specimens (7 mm × 7 mm) were fixed onto the bottom of the inserts. Since dexamethasone is a widely recognized potent anti-inflammatory agent, a positive-control group treated with 10 μM dexamethasone (DEX) was set for reference. A total of 600 μL and 200 μL of fresh complete medium were added to the lower and upper Transwell chambers, respectively. All culture media were supplemented with 0.1% (*v*/*v*) Tween 80, and 1 μg/mL LPS was introduced to all groups except the blank control group. Following incubation at 37 °C in a 5% CO_2_ incubator for 24 h, cell culture supernatants were harvested. A double-antibody sandwich ELISA was used to measure the optical density (OD) at 450 nm, and the inhibition rates of inflammatory cytokines were calculated accordingly.

### 2.12. Cytocompatibility Assessment

#### 2.12.1. Cell Viability Assessment on Pure Fiber Membranes

To assess the biosafety and cell viability of the base fiber membrane, human umbilical vein endothelial cells (HUVECs) were seeded onto drug-free PCL/PEO-PVP fiber membranes at a density of 10^5^ cells/mL and cultured in a humidified incubator at 37 °C with 5% CO_2_ for 1 d, 3 d and 5 d. Live/Dead staining was performed using a Calcein-AM/PI double staining kit (MKBio, Shanghai, China) with an incubation duration of 20 min. An inverted fluorescence microscope (LHM100CB-1, Nikon, Tokyo, Japan) was employed to visualize live and dead cells on the fiber membranes.

#### 2.12.2. Cell Viability Assessment on Dual-Drug Sequential Release Fiber Membranes

Curcumin (CUR) is an intrinsically fluorescent substance that emits strong fluorescence under specific excitation wavelengths [35]. Conventional Calcein-AM/PI Live/Dead staining employs 488 nm blue-light excitation, which excites not only Calcein but also CUR. This generates substantial background fluorescence and obscures the fluorescent signals of live cells (Figure S1). Therefore, a modified Live/Dead staining protocol was adopted. Cells were seeded and cultured on fiber membranes following the procedure described in Section 2.12.1. Before fluorescence staining, the membranes were transferred into new culture plates and treated with trypsin for 5 min. The membranes were gently agitated to detach adherent cells and then removed, and fresh complete medium was supplemented to terminate digestion. The harvested cells were centrifuged, rinsed, and resuspended in 600 μL of fresh medium [36]. Half of the cell suspension was seeded into a new 24-well plate for Live/Dead staining at 6 h after re-seeding (at which point cells had reattached but not yet proliferated) [37]. The remaining suspension was split equally and dispensed into three wells of a 96-well plate for the CCK-8 assay (10% CCK-8 reagent, 1 h incubation), followed by OD measurement.

#### 2.12.3. Biosafety Evaluation of Membrane Extracts

The CCK-8 assay was adopted to assess the biosafety of extractables released from fiber membranes. The fiber membranes were sterilized via short-duration UV irradiation (to prevent drug degradation induced by prolonged UV exposure) and immersed in complete medium for 48 h to prepare membrane extraction medium. HUVECs were seeded in 96-well plates at a density of 5 × 10^4^ cells/mL (100 μL per well) and incubated for 4 h to achieve cell attachment. The original medium was replaced with either fresh complete medium (blank control) or membrane extraction medium, and cells were incubated at 37 °C with 5% CO_2_. After 1 d, 3 d and 5 d of culture, 10% CCK-8 reagent was added to each well. The plates were incubated in darkness for 1 h, and the OD values were measured at 450 nm using a microplate reader [38].

### 2.13. Statistical Analysis

All data are expressed as mean ± standard deviation (SD). Statistical analysis was performed using Origin 2024 software(OriginLab Corporation, Northampton, MA, USA). Quantitative data were obtained from at least three independent replicates. Statistical significance was defined as * *p* < 0.05, ** *p* < 0.01, *** *p* < 0.001.

## 3. Results and Discussion

### 3.1. Analysis of the Effects of Key Process Parameters on Coaxial Electrospinning

The forming quality of coaxial-electrospun fiber membranes was evaluated through both macroscopic and microscopic morphologies, and 100 randomly selected fibers from SEM images were measured for diameter statistics.

**Figure 3:**
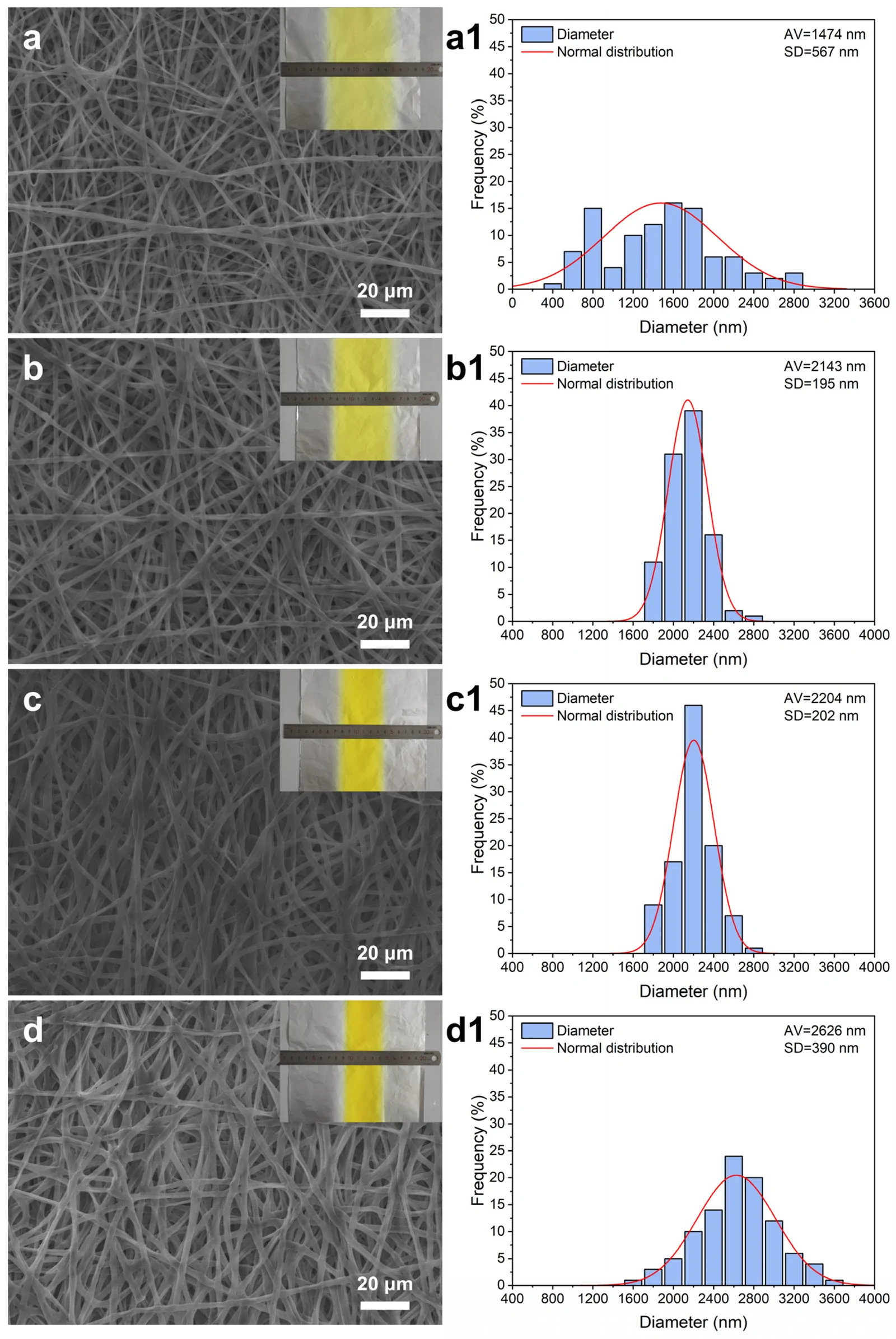
SEM images and fiber diameter distribution of coaxial electrospun membranes prepared with different total core solution concentrations. (**a**,**a1**) 2% (*w*/*v*); (**b**,**b1**) 4% (*w*/*v*); (**c**,**c1**) 6% (*w*/*v*); (**d**,**d1**) 8% (*w*/*v*). The preferred parameters are 4% (*w*/*v*) and 6% (*w*/*v*).

Total core solution concentration (A): The concentration of the core solution directly regulates viscosity matching between the two liquid phases and plays a critical role in the spinnability and core–shell structural integrity of coaxial fibers [39,40]. As shown in Figure 3, at a core concentration of 2% (*w*/*v*), the solution viscosity is excessively low. The coaxial jet bears uneven stress and inconsistent elongation under electric field stretching, resulting in poor jet stability and inhomogeneous drawing. Microscopically, the resulting fibers exhibit substantial diameter fluctuations and extremely broad diameter distribution. Macroscopically, the membrane presents the widest width, indicating uneven electric field stretching and excessive jet divergence. When the core concentration increases to 4% (*w*/*v*) and 6% (*w*/*v*), the solution viscosity satisfies the mechanical requirements of electrospinning drawing, enabling synchronous elongation with the shell solution and stable jet formation without breakage. Continuous core–shell fibers with smooth morphology, uniform diameter, full core filling and intact shell encapsulation are obtained. Fibers prepared at 4% (*w*/*v*) show a small average diameter and low diameter variation, while the 6% (*w*/*v*) group exhibits a narrower macroscopic width, indicating enhanced electric field stretching. At a core concentration of 8% (*w*/*v*), the excessively high viscosity reduces fluidity and causes rheological mismatch with the PCL shell solution, decreasing overall jet ductility and hindering sufficient refinement by the electric field. The resultant fibers display a markedly increased average diameter, local bulges and irregular protrusions, with the narrowest and uneven membrane surface.

**Figure 4:**
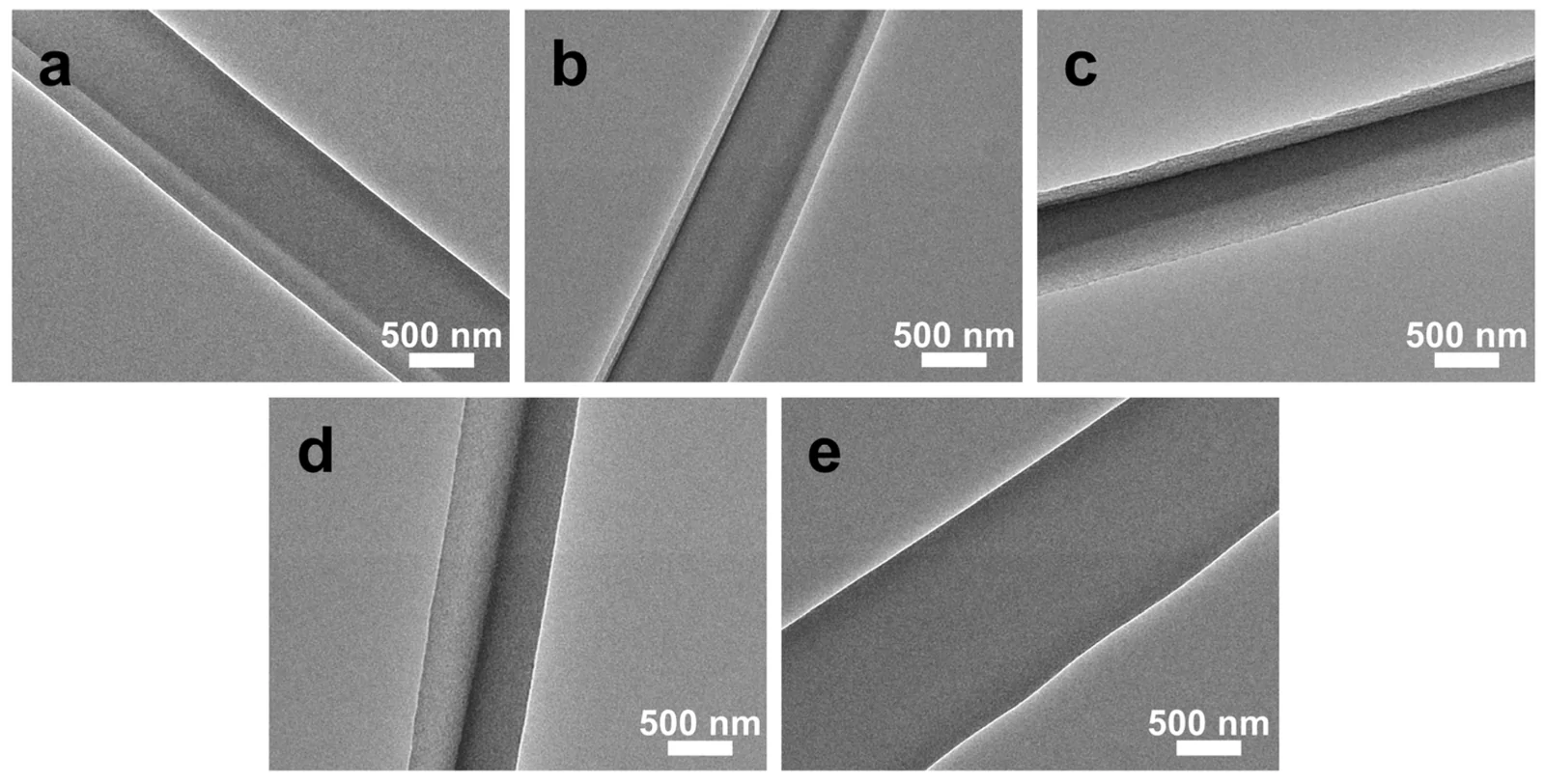
TEM images of coaxial electrospun membranes prepared with different core-to-shell flow rate ratios. (**a**) 1:3; (**b**) 1:4; (**c**) 1:5; (**d**) 1:6; (**e**) 1:7. The preferred parameters are 1:4,1:5 and 1:6.

Core-to-shell flow rate ratio (B): The core-to-shell flow rate ratio directly controls the volume ratio of inner and outer fluids, strongly affecting core–shell integrity and drug loading performance [41]. As shown in Figure 4, at a ratio of 1:3, excessive core flow relative to the shell flow leads to an overly thick core and insufficient shell encapsulation, with partial core breakthrough and compromised layered structure. At ratios of 1:4, 1:5 and 1:6, well-matched fluid ratios produce smooth, regular fibers with complete core encapsulation and clear core–shell boundaries, without eccentric core or shell rupture defects; nevertheless, the shell layer is relatively thin at 1:4, and the core becomes overly slender at 1:6. At a ratio of 1:7, insufficient core flow results in core discontinuity, and a distinct core–shell structure can no longer be observed.

**Figure 5:**
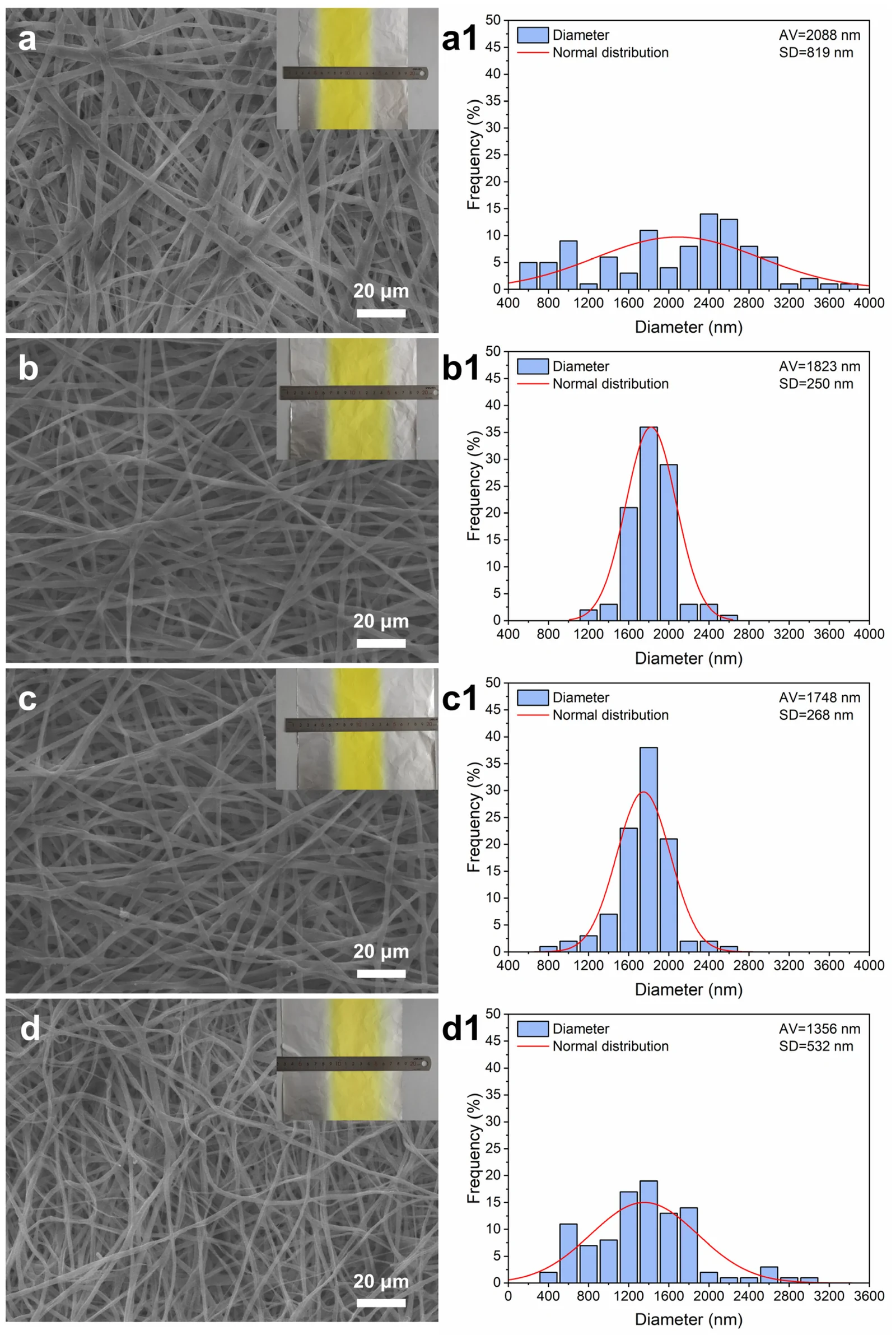
SEM images and fiber diameter distribution of coaxial electrospun membranes prepared with different electrospinning voltages. (**a**,**a1**) 10 kV; (**b**,**b1**) 12 kV; (**c**,**c1**) 14 kV; (**d**,**d1**) 16 kV. The preferred parameters are 12 kV and 16 kV.

Electrospinning voltage (C): Applied voltage governs jet stretching behavior and strongly determines fiber morphology and quality [42]. As shown in Figure 5, at 10 kV, the electric field strength is insufficient for adequate jet elongation, producing generally thick fibers with broad diameter distribution, local bulges and irregular deformations, accompanied by a wider macroscopic membrane. At 12 kV and 14 kV, moderate electric field force sustains stable jet formation and yields smooth, defect-free, uniform fibers; the 14 kV group shows a narrower membrane width, reflecting stronger electric field stretching. At 16 kV, excessive electric field intensity induces violent jet oscillation, forming curved, irregular fibers with numerous thin segments; the membrane becomes wider and thinner with surface particles and protrusions.

**Figure 6:**
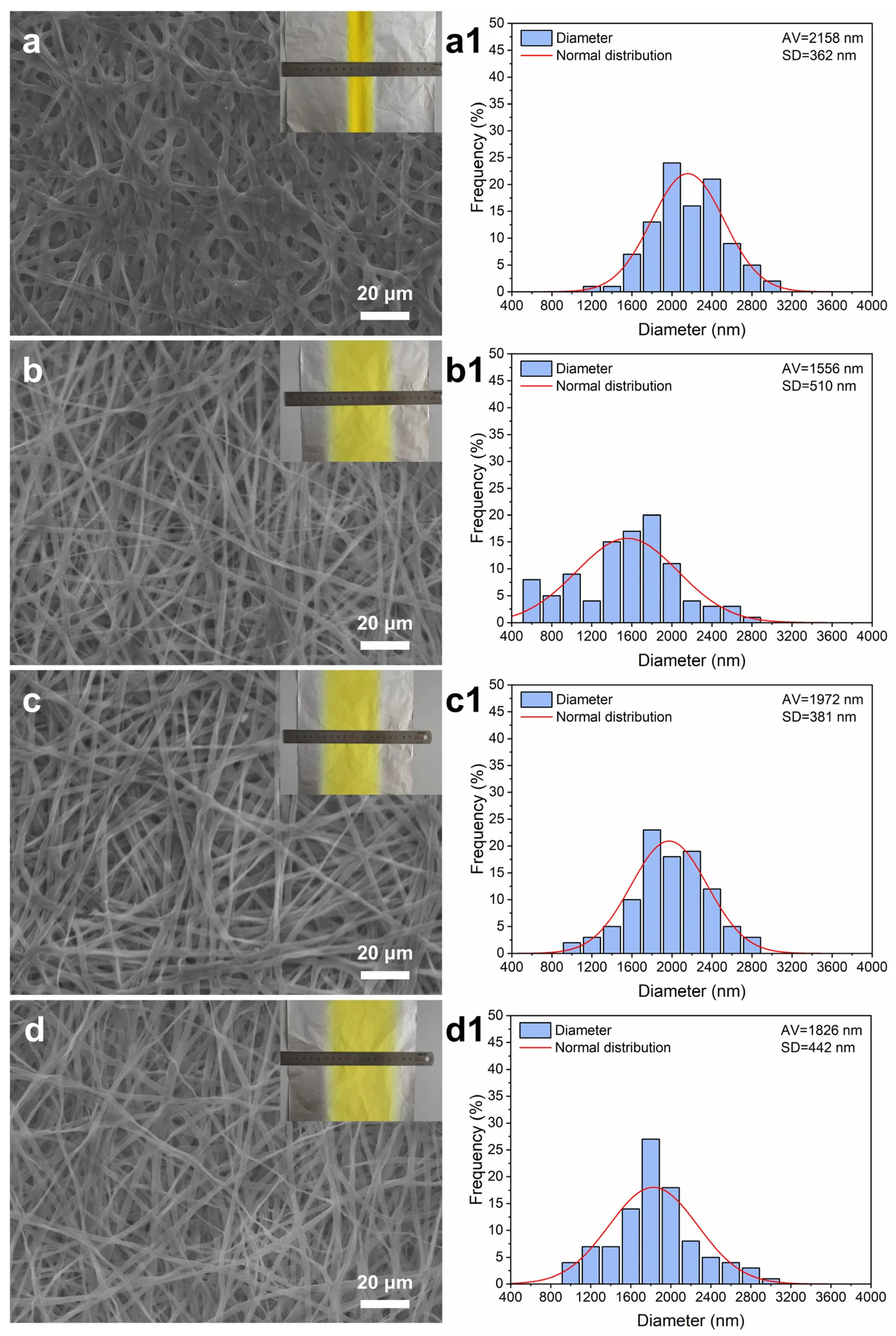
SEM images and fiber diameter distribution of coaxial electrospun membranes prepared with different collecting distances. (**a**,**a1**) 12 cm; (**b**,**b1**) 15 cm; (**c**,**c1**) 18 cm; (**d**,**d1**) 21 cm. The preferred parameter is 18 cm.

Collecting distance (D): The collecting distance determines jet flight time and solvent evaporation efficiency, significantly influencing fiber morphology and diameter uniformity [43]. As shown in Figure 6, at 12 cm, the short flight time results in incomplete solvent evaporation, causing fiber adhesion, coarse fibers and high diameter variation; the membrane exhibits an extremely narrow width due to intensified short-range electric field stretching. At 15 cm, solvent evaporation improves but electric field effects still cause uneven fiber diameters. At 18 cm, optimal electric field stretching produces smooth, non-adhesive, uniform continuous fibers. At 21 cm, electric field strength decays with distance, reducing stretching force while extending flight time. Although the average fiber diameter decreases, jet stability deteriorates, fiber breakage occurs, and overall morphological regularity declines, corresponding to the widest macroscopic membrane caused by reduced electric field confinement and prolonged divergent jet flight.

### 3.2. Orthogonal Test Results

Based on the parameter analysis presented in Section 3.1, suitable factor levels were selected as follows:

- Total core solution concentration (A): 4% (*w*/*v*), 5% (*w*/*v*), 6% (*w*/*v*)
- Core-to-shell flow rate ratio (B): 1:4, 1:5, 1:6
- Electrospinning voltage (C): 12 kV, 13 kV, 14 kV
- Collecting distance (D): 16 cm, 18 cm, 21 cm

The detailed orthogonal experimental layout is listed in Table 1.

Nine groups of specimens were characterized. The average values of each indicator and the corresponding comprehensive scores are summarized in Table 2 (See Table S1 for details). The optimal parameter combination was identified as A_1_B_1_C_3_D_2_. Range analysis results (Table 3) reveal that the influencing sequence of the four factors follows C > D > B > A. Factor C dominates the overall performance, followed by D, A and B in sequence.

### 3.3. FTIR Analysis

**Figure 7:**
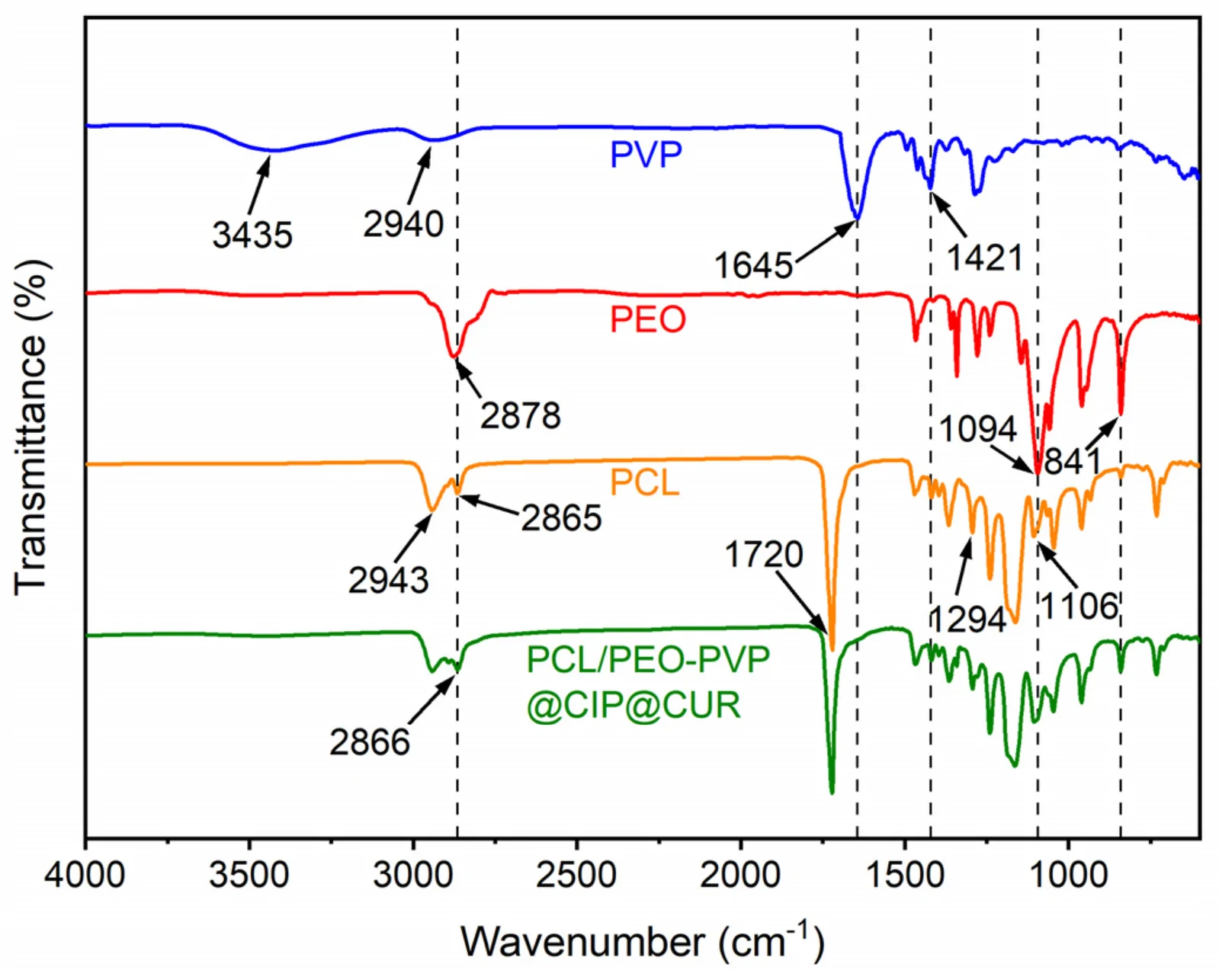
FTIR spectra of neat PVP, neat PEO, neat PCL, and PCL/PEO-PVP@CIP@CUR coaxial electrospun fibrous membranes.

Fourier-transform infrared spectroscopy (FTIR) was adopted to characterize the chemical structure and intermolecular interactions of PCL/PEO-PVP@CIP@CUR coaxial fiber membranes, as shown in Figure 7. Neat PVP displayed a broad absorption band at 3435 cm^−1^ (O–H stretching vibration) and a characteristic peak at 1645 cm^−1^ (C=O stretching vibration of the pyrrolidone ring). Neat PEO exhibited a typical C–O–C ether stretching vibration peak at 1094 cm^−1^. Neat PCL possessed a prominent ester C=O stretching peak at 1720 cm^−1^, along with methylene C–H stretching vibration peaks at 2943 cm^−1^ and 2865 cm^−1^. The main characteristic peaks of PCL, PEO and PVP were all clearly observed in the spectrum of PCL/PEO-PVP@CIP@CUR composite fibers, accompanied by slight peak shifts and intensity variations. These findings confirm the successful incorporation of each polymeric component, and the primary backbone chemical structure of the matrix remains intact.

### 3.4. Hydrophilicity

**Figure 8:**
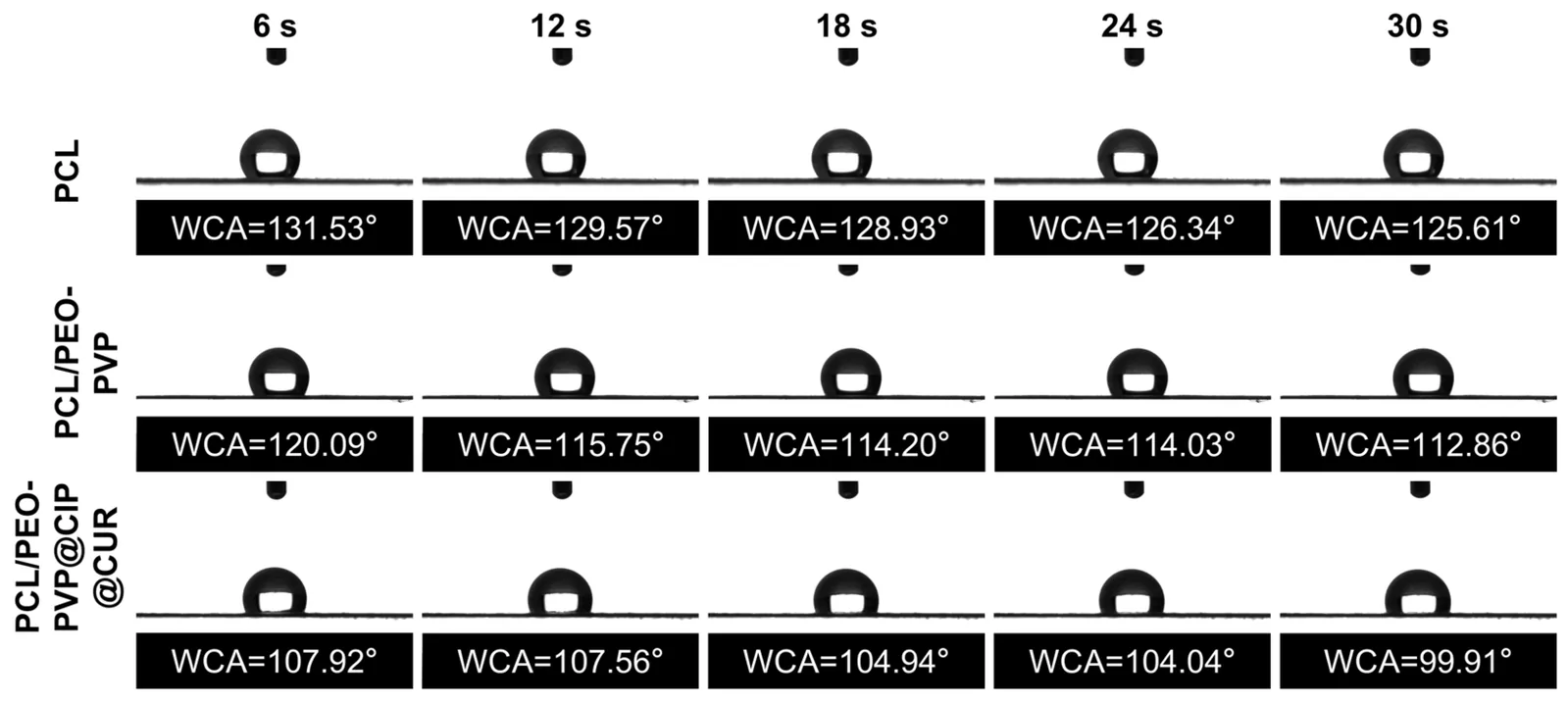
Dynamic water contact angle evolution of electrospun fibrous membranes at different time points.

Dynamic water contact angle measurements were conducted to assess the surface wettability of three groups of fiber membranes (Figure 8). The neat PCL monolithic fiber membrane showed an initial water contact angle of 131.53°, which remained at 125.61° after 30 s, demonstrating obvious hydrophobicity. After fabricating the coaxial core–shell structure with hydrophilic PEO and PVP, the initial water contact angle of the PCL/PEO-PVP membrane decreased to 120.09° and reached 112.86° at 30 s. The surface wettability was improved, yet the membrane remained predominantly hydrophobic. Following loading of CIP and CUR, the PCL/PEO-PVP@CIP@CUR coaxial fiber membrane possessed an initial water contact angle of 107.92°, which declined to 99.91° after 30 s with enhanced overall hydrophilicity. Nevertheless, the surface property was still dominated by the hydrophobic PCL shell layer.

These findings confirm that the continuous outer PCL shell governs surface wettability, guaranteeing structural stability and tunable long-term degradation. The gradual reduction of contact angle over time reflects the slow contribution of inner hydrophilic components and avoids excessively rapid water penetration.

### 3.5. Degradation Performance

**Figure 9:**
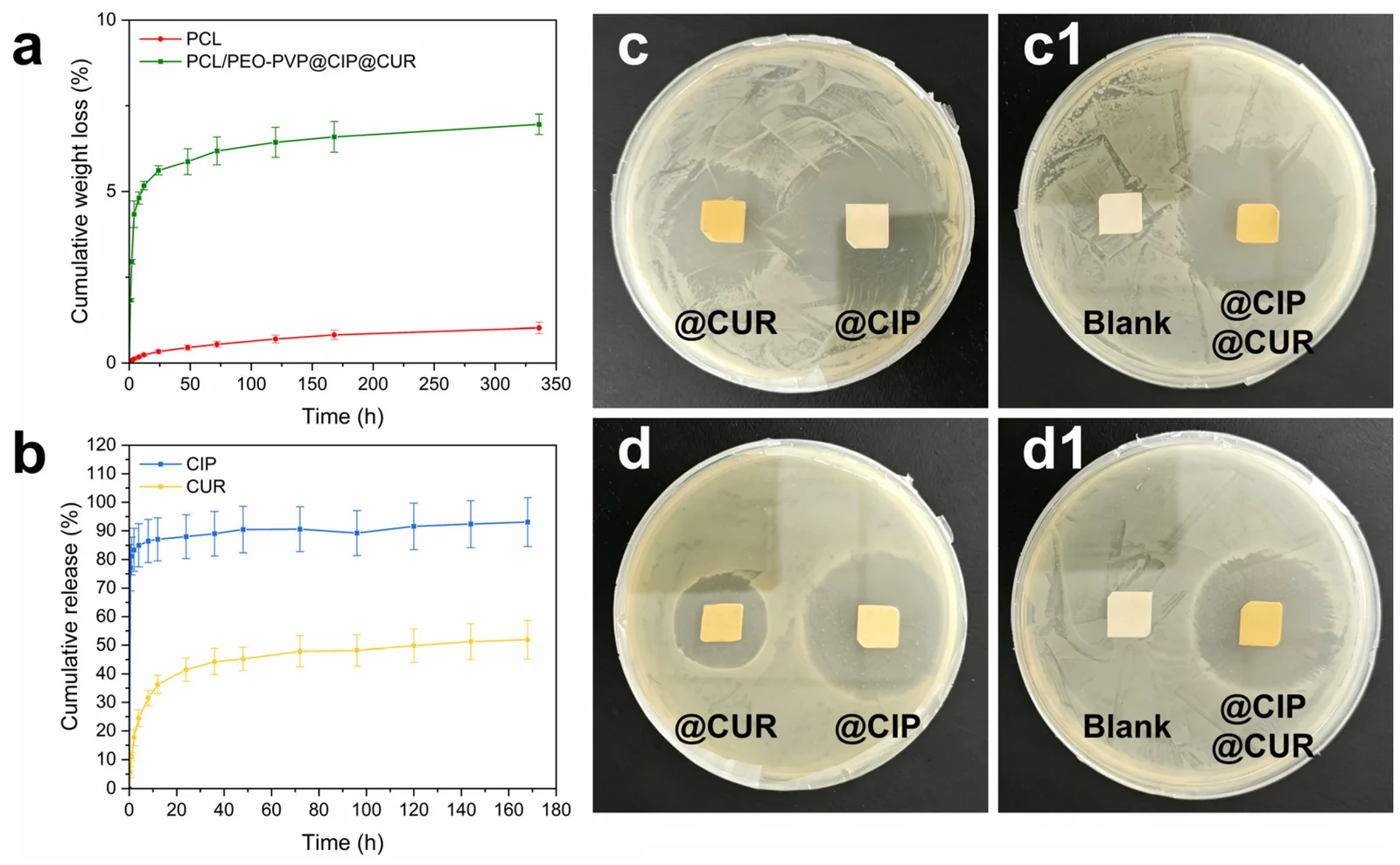
(**a**) In vitro cumulative weight loss curves of neat PCL and PCL/PEO-PVP@CIP@CUR electrospun fibrous membranes; (**b**) In vitro cumulative release profiles of CIP and CUR from PCL/PEO-PVP@CIP@CUR electrospun fibrous membranes; (**c**,**c1**) Photographs of *S. aureus* inhibition zones of different fibrous membrane samples after 24-h incubation; (**d**,**d1**) Photographs of *E. coli* inhibition zones of different fibrous membrane samples after 24-h incubation.

The in vitro cumulative mass loss profiles of neat PCL fiber membranes and PCL/PEO-PVP@CIP@CUR coaxial fiber membranes are presented in Figure 9a. Neat PCL membranes displayed an extremely slow degradation rate, with a total mass loss of merely 1.025% over two weeks, indicating gradual and homogeneous degradation. This phenomenon originates from the intrinsic hydrophobic long-chain structure of the PCL polyester matrix, which hinders hydrolysis and sustains long-term structural integrity. The PCL/PEO-PVP@CIP@CUR core–shell fiber membranes underwent an initial rapid mass loss stage (5.17% mass loss at 12 h), followed by a slower degradation rate and a stable plateau, reaching a final total mass loss of 6.96% at two weeks. The initial rapid weight loss mainly stems from the dissolution and diffusion of hydrophilic PEO, PVP and water-soluble drugs within the core layer, instead of rapid hydrolysis of the PCL shell. The subsequent steady degradation verifies that the outer PCL shell serves as an effective barrier to retard the overall degradation rate of the matrix.

### 3.6. In Vitro Drug Release Study

In vitro drug release profiles were acquired to characterize the release kinetics of the dual-drug-loaded system (Figure 9b), verifying sequential biphasic release of CIP and CUR from PCL/PEO-PVP@CIP@CUR coaxial fiber membranes.

Despite the dense hydrophobic PCL outer shell, ciprofloxacin displays evident initial burst release within 30 min. Ciprofloxacin hydrochloride is highly hydrophilic [44] and readily soluble in aqueous media. Once a small volume of water penetrates the PCL shell, the inner hydrophilic PEO-PVP matrix swells rapidly and establishes an osmotic pressure gradient, drawing extra water into the fiber core. Highly water-soluble CIP rapidly diffuses through the swollen matrix and across the thin PCL shell, resulting in fast initial release.

In comparison, curcumin possesses a hydrophobic aromatic backbone with negligible hydrophilic moieties, exhibiting prominent lipophilicity [45] and hindering rapid permeation through the outer shell. Moreover, curcumin forms intermolecular hydrogen bonds with PVP to generate stable complexes immobilized within the PEO-PVP polymeric network, which substantially elevates diffusion resistance [46,47]. The hydrophobic PCL shell further retards water ingress and curcumin diffusion, facilitating sustained and delayed long-term CUR release.

In brief, the osmotic pressure built up inside the hydrophilic core phase triggers the rapid early release of CIP. In contrast, the inherent strong hydrophobicity of CUR, polymer molecular entanglement, and the barrier effect of the PCL shell collectively achieve markedly delayed sustained release of CUR.

### 3.7. Antibacterial Performance

The antibacterial activities of dual-drug coaxial fiber membranes were estimated by the “antibacterial zone” method with 24 h incubation [48], as shown in Figure 9.

Among the single-drug groups, the inhibition zones formed by PCL/PEO-PVP@CIP membranes were remarkably larger than those of PCL/PEO-PVP@CUR membranes against both *Staphylococcus aureus* and *Escherichia coli* (Figure 9c,d). This reveals that CIP dominates the early-stage antibacterial performance of the coaxial fibers, which can be ascribed to differences in pharmacological activity or drug release profiles. The blank drug-free membrane (PCL/PEO-PVP) showed no antibacterial activity. The inhibition zone of the PCL/PEO-PVP@CIP@CUR dual-drug membrane was comparable to that of the PCL/PEO-PVP@CIP membrane (Figure 9(c1,d1)), demonstrating that the early antibacterial efficacy of the dual-drug formulation is at least equivalent to that of the CIP single-drug formulation.

### 3.8. Anti-Inflammatory Performance

**Figure 10:**
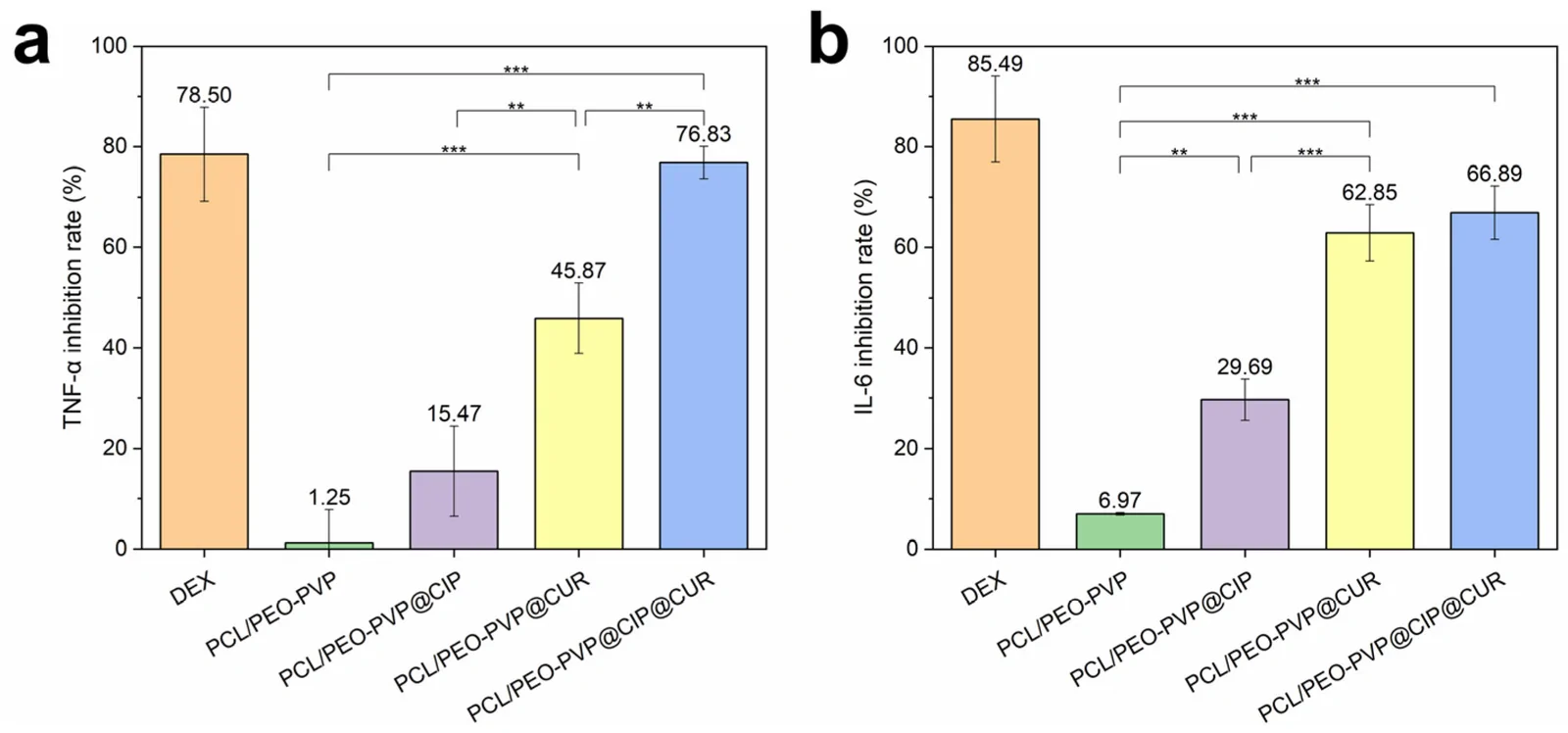
Inflammatory cytokine inhibition rates of various electrospun fibrous membranes determined by ELISA. (**a**) TNF-α; (**b**) IL-6. (* *p* < 0.05, ** *p* < 0.01, *** *p* < 0.001).

ELISAs were conducted to quantitatively compare the anti-inflammatory effects of different membrane groups via detection of key inflammatory cytokines, TNF-α and IL-6 (Figure 10). The dexamethasone (DEX) group exerted the strongest inhibitory effect, whereas the drug-free PCL/PEO-PVP membrane showed negligible cytokine inhibition. The PCL/PEO-PVP@CIP membrane achieved moderate anti-inflammatory activity, implying that the high initial CIP concentration arising from rapid burst release can partially suppress inflammatory responses [49]. The PCL/PEO-PVP@CUR membrane displayed stronger cytokine inhibition than the PCL/PEO-PVP@CIP membrane, verifying the remarkable anti-inflammatory property of CUR [50,51]. The PCL/PEO-PVP@CIP@CUR dual-drug membrane yielded even higher inhibition rates, particularly for TNF-α. These findings confirm that loading therapeutic drugs imparts anti-inflammatory activity to fiber membranes, and the dual-drug membrane presents improved anti-inflammatory performance in comparison with single-drug formulations.

### 3.9. Cytocompatibility Evaluation

**Figure 11:**
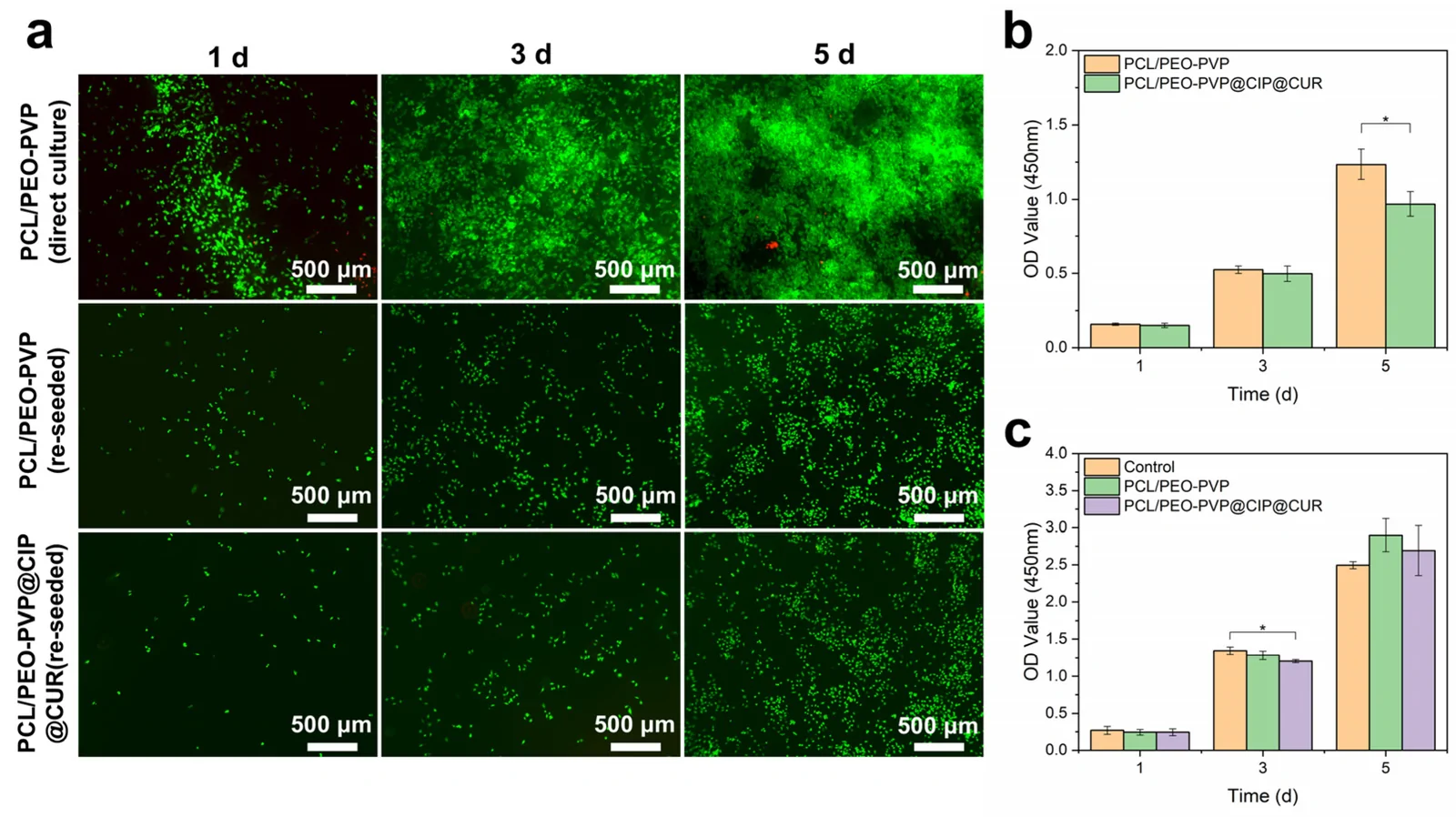
HUVEC adhesion, spreading and proliferation characteristics for biocompatibility assessment of various electrospun fibrous membranes. (**a**) Fluorescence micrographs of HUVECs cultured on membranes (green: live cells, red: dead cells); (**b**) CCK-8 viability of harvested HUVECs after co-culture with fibrous membranes and re-seeding for 6 h; (**c**) CCK-8 cytotoxicity results of HUVECs cultured in membrane extracts. (* *p* < 0.05, ** *p* < 0.01, *** *p* < 0.001).

Human umbilical vein endothelial cell (HUVEC) adhesion and proliferation assays were conducted to assess the biocompatibility of PCL/PEO-PVP@CIP@CUR dual-drug fiber membranes (Figure 11a). To avoid severe interference originating from the intrinsic fluorescence of CUR, cell adhesion experiments were first carried out on drug-free PCL/PEO-PVP base membranes. On day 1, cells were sparsely distributed yet successfully adhered to the fiber surface. On day 3, the cell population increased evidently with spreading morphology, confirming favorable cell attachment and spreading. By day 5, cells proliferated extensively and interconnected, exhibiting typical polygonal morphology, which verifies the excellent cytocompatibility of the base polymeric matrix.

After characterizing the base membranes, HUVECs were seeded onto PCL/PEO-PVP@CIP@CUR dual-drug membranes, with drug-free PCL/PEO-PVP membranes set as the control group. Cells were harvested and re-seeded before staining, and cell proliferation was evaluated accordingly. On day 1, cells were evenly distributed with spindle shapes and pseudopodium formation, indicating normal cell attachment without anoikis for both groups. On day 3, further cell migration and spreading were observed in both groups. On day 5, obvious cell proliferation was confirmed in all groups, demonstrating preserved cell adhesion function. CCK-8 OD data (Figure 11b) revealed slightly lower cell viability in the dual-drug group at day 5, suggesting a minor effect of the loaded drugs on cell proliferation.

A CCK-8 assay was further conducted to evaluate the cytotoxicity of membrane extracts (Figure 11c). The cell viability measured for the PCL/PEO-PVP extract group was nearly consistent with that of the pure medium control group, whereas the PCL/PEO-PVP@CIP@CUR extract group only exhibited a slight decline in cell viability at day 3 compared with the drug-free group.

## 4. Conclusions

In this work, a dual-drug coaxial fiber membrane designated PCL/PEO-PVP@CIP@CUR co-loaded with CIP and CUR was fabricated via coaxial electrospinning, aiming for antibacterial and anti-inflammatory applications in vascular stents. The outer shell of the coaxial fibers is made of PCL, while the core matrix comprises PEO and PVP.

Firstly, the optimal fabrication parameters were identified through orthogonal experiments. Chemical characterizations verified that the backbone chemical structure of the polymer matrix remained intact after electrospinning and that all polymeric components were successfully incorporated. TEM observations confirmed the successful preparation of core–shell structured fibers. Combined dynamic water contact angle tests and CUR release profiles further proved that the inner core phase was effectively encapsulated by the outer PCL shell. Mass loss measurements demonstrated that the PCL shell is capable of sustaining long-term structural stability. The drug release profiles revealed a sequential delivery pattern: rapid initial drug release to achieve immediate antibacterial activity, followed by sustained slow release for prolonged anti-inflammatory treatment. Antibacterial and anti-inflammatory assays suggested that CIP and CUR exert distinct and irreplaceable functions. Cellular experiments validated the favorable biocompatibility of the dual-drug coaxial membrane, which supports stable adhesion and proliferation of endothelial cells.

The PCL/PEO-PVP@CIP@CUR fiber membrane offers a promising new strategy to satisfy clinical requirements for controlling acute early infection and regulating long-term inflammation following vascular stent implantation.

## Figures and Tables

**Table 1:** Layout of orthogonal experiment.

Group	Factors			
	A (*w*/*v*)	B (/)	C (kV)	D (cm)
1	4% (1)	1:4 (1)	12 (1)	16 (1)
2	4% (1)	1:5 (2)	14 (3)	18 (2)
3	4% (1)	1:6 (3)	13 (2)	20 (3)
4	5% (2)	1:4 (1)	14 (3)	18 (2)
5	5% (2)	1:5 (2)	13 (2)	20 (3)
6	5% (2)	1:6 (3)	12 (1)	16 (1)
7	6% (3)	1:4 (1)	13 (2)	20 (3)
8	6% (3)	1:5 (2)	14 (3)	16 (1)
9	6% (3)	1:6 (3)	12 (1)	18 (2)

**Table 2:** Measured indicators and comprehensive scores for each orthogonal experimental group.

Group	*a* (%)	*b* (MPa)	*c* (/)	*d* (%)	*y*
1	30.7725	4.0753	0.32209	0.6139	4.54
2	23.9570	6.1197	0.3625	0.5175	6.50
3	29.0310	3.8857	0.33643	0.4338	3.49
4	21.9146	2.7610	0.16336	0.5731	5.73
5	35.8845	3.2023	0.2749	0.5228	2.76
6	29.3732	3.1270	0.27165	0.3822	2.94
7	26.9267	2.5817	0.32625	0.5599	3.57
8	14.3535	2.5200	0.45463	0.4502	4.03
9	26.3434	3.1513	0.35299	0.3615	2.78

**Table 3:** Range analysis of evaluation indicators from orthogonal experiment.

Parameter	Factors			
	A	B	C	D
*K*_1_	14.53	13.83	10.26	11.51
*K*_2_	11.43	13.29	9.82	15
*K*_3_	10.37	9.21	16.25	9.82
k_1_	4.84	4.61	3.42	3.84
k_2_	3.81	4.43	3.27	5
k_3_	3.46	3.07	5.42	3.27
*R*	1.39	1.54	2.14	1.73
Optimal Combination	A_1_B_1_C_3_D_2_			
Factor Influence Order	C > D > B > A			

## Data Availability

The data that support the findings of this study are available on request from the corresponding authors. The data are not publicly available due to privacy.

## Supplementary Materials

The following supporting information can be downloaded at: https://www.mdpi.com/article/10.3390/biomimetics11080599/s1, Figure S1: Background fluorescence generated by CUR in fibrous membranes under 488 nm excitation interferes with live cell fluorescent signals; Table S1: Normalized indicators and comprehensive scores from orthogonal experiments.
