# Supplementary material for: Preparation and Characterization of PCL/PEO-PVP ECM-Mimicking Coaxial Electrospun Membranes Loaded with Ciprofloxacin and Curcumin for Sequential Dual-Drug Release
Source: Biomimetics (Basel). 2026 Aug 21;11(8):599. doi: 10.3390/biomimetics11080599 (PMC13509903; doi:10.3390/biomimetics11080599)
Supplement: Supplementary file 1 [file biomimetics-11-00599-s001.zip › biomimetics-4486667-supplementary.pdf]

Table S1. Normalized indicators and comprehensive scores from orthogonal experiments.

Group	*a* (%)	y_a	*b* (MPa)	y_b	*c* (/)	y_c	*d* (%)	y_d	*y*
1	30.7725	0.24	4.0753	0.43	0.32209	0.46	0.6139	1.00	4.54
2	23.9570	0.55	6.1197	1.00	0.3625	0.32	0.5175	0.62	6.50
3	29.0310	0.32	3.8857	0.38	0.33643	0.41	0.4338	0.29	3.49
4	21.9146	0.65	2.7610	0.07	0.16336	1.00	0.5731	0.84	5.73
5	35.8845	0.00	3.2023	0.19	0.2749	0.62	0.5228	0.64	2.76
6	29.3732	0.30	3.1270	0.17	0.27165	0.63	0.3822	0.08	2.94
7	26.9267	0.42	2.5817	0.02	0.32625	0.44	0.5599	0.79	3.57
8	14.3535	1.00	2.5200	0.00	0.45463	0.00	0.4502	0.35	4.03
9	26.3434	0.44	3.1513	0.18	0.35299	0.35	0.3615	0.00	2.78

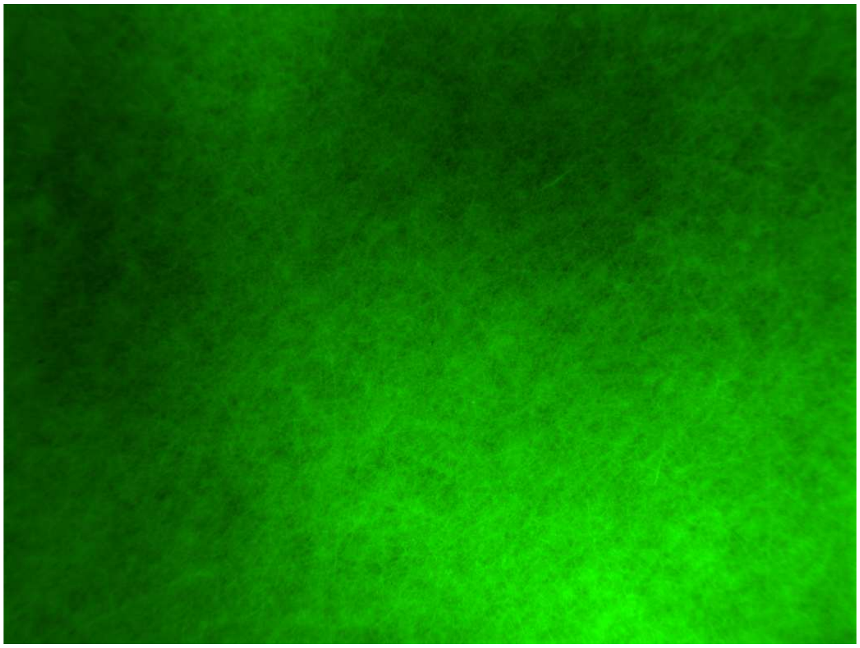

Figure S1. Background fluorescence generated by CUR in fibrous membranes under 488 nm excitation interferes with live cell fluorescent signals.
